# Progesterone: The Key Factor of the Beginning of Life

**DOI:** 10.3390/ijms232214138

**Published:** 2022-11-16

**Authors:** Carlo Bulletti, Francesco Maria Bulletti, Romualdo Sciorio, Maurizio Guido

**Affiliations:** 1 Extra Omnes, Assisted Reproductive Technology, ART Center, Via Gallinelli, 8, 47841 Cattolica, Italy; 2 Department of Obstetrics, Gynecology, and Reproductive Science, Yale University, New Haven, CT 06510, USA; 3 Department Obstetrics and Gynecology, University Hospital of Vaud, 1011 Lausanne, Switzerland; 4 Edinburgh Assisted Conception Programme, Royal Infirmary of Edinburgh, Edinburgh EH16 4SA, UK; 5 Obstetrics and Gynecology Unit, Department of Life, Health and Environmental Sciences, University of L’Aquila, 67100 L’Aquila, Italy

**Keywords:** progesterone, progesterone supplementation, luteal phase support, implantation

## Abstract

Progesterone is the ovarian steroid produced by the granulosa cells of follicles after the LH peak at mid-cycle. Its role is to sustain embryo endometrial implantation and ongoing pregnancy. Other biological effects of progesterone may exert a protective function in supporting pregnancy up to birth. Luteal phase support (LPS) with progesterone is the standard of care for assisted reproductive technology. Progesterone vaginal administration is currently the most widely used treatment for LPS. Physicians and patients have been reluctant to change an administration route that has proven to be effective. However, some questions remain open, namely the need for LPS in fresh and frozen embryo transfer, the route of administration, the optimal duration of LPS, dosage, and the benefit of combination therapies. The aim of this review is to provide an overview of the uterine and extra-uterine effects of progesterone that may play a role in embryo implantation and pregnancy, and to discuss the advantages of the use of progesterone for LPS in the context of Good Medical Practice.

## 1. Introduction

Progesterone (from progestin pro “for” and gest “pregnancy”) is natural progestin, Pregn-4-ene-3, 20-dione or P4, and is the most abundant hormone produced by the gonads. It is synthesized primarily by the corpus luteum of the ovary, by the adrenal cortex and by the placenta in case of pregnancy. In men, progesterone is produced by the testes and adrenal cortex. Its presence is a prerequisite for embryo implantation whereas its absence causes pregnancy loss [1,2,3]. In fact, luteal phase defects, i.e., a short or inadequate luteal phase associated with inadequate progesterone production, reduce the chances of a successful pregnancy. Progesterone also exerts significant extra-reproductive actions via multiple non-genomic signaling pathways. These functions include immunomodulation [4,5], utero-relaxation [6], inhibition of cholesterol biosynthesis [7], and neuroprotection [8,9].

Before successful implantation of a blastocyst in the pre-decidualized endometrium, morphological and biochemical changes of both the embryo and the endometrium must be in synchrony. The embryo-endometrium synchrony occurs internally and externally via vesicles and/or metabolic products that are delivered to the virtual cavity of the uterus by both the blastocyst and the endometrium [10,11,12,13,14,15]. 

The use of progesterone to treat luteal phase (LP) defects was proposed by the pioneer of gynecological endocrinology, Georgeanna Seegar Jones, who later also introduced the “Window of Implantation” (WOI) concept [16]. Subsequently, Lessey [17] reported that progesterone supplementation during the LP results in morphological changes caused by the priming of estrogens in the proliferative phase of the endometrium [18]. However, the Noyes criteria do not seem to be sufficiently accurate to identify LP defects due to the variability of at least 48 h in dating the LP [19].

When the proliferation of the epithelial cells of the endometrial glands of the inner surface of the lumen cavity is stimulated by estrogen [20], the progesterone produced by granulosa cells induces gland glycogen accumulation. Consequently, the epithelial cells become pluri-stratified and stromal cells become decidual (i.e., larger and round-shaped), and they acquire the ability to produce proteins. At this stage, the microvascular supply in the functional endometrium changes considerably, both morphologically and functionally, to enable embryo implantation. Progesterone is the main actor in all these phases. 

From a biochemical point of view, the most dramatic changes are detected in the decidual cells that contain basement membranes during mid-secretory phase of the endometrial cycle. In fact, in this phase, prolactin, proteoglycans, laminin, and collagen IV are detectable by immunohistochemistry [21]. The mid-secretory phase is also characterized by the appearance of pinopodes on the surface of the endometrium [22]. Unfortunately, pinopodes are detectable only by electron microscopy, which is time-consuming and requires a mock cycle, and is thus rarely used in clinical practice. More recently, transcriptomic evaluation was proposed as an alternative to histology to evaluate endometrial adequacy for embryo nidation [23,24].

Assisted reproductive technologies (ART) are increasingly being used to assist couples to have a family. In ART programs, less than one in three cases of treatment for infertility involving an embryo results in a live birth [25]. Implantation failure of euploid embryos is one of the most important limiting factors in ART success and remains a “black hole” in our knowledge. To date, LPS with progesterone is the only safe and effective treatment able to improve embryo implantation [26], notwithstanding variable outcomes depending on cycle type (fresh or frozen) and on progesterone plasma levels during the LP [27]. While the efficacy of progesterone in fresh embryo transfer (ET) is now well established [26], the optimal LPS in frozen embryo transfer (FET) has yet to be established, particularly in terms of indication to treatment [27], route of administration [28], and treatment duration [29].

Frozen embryo transfer is an important option for single ET and can limit the risk of ovarian hyperstimulation syndrome (OHSS), which is a serious adverse event of ovarian stimulation [30]. Recent evidence indicates that circulating progesterone during hormone replacement therapy (HRT) may affect ongoing pregnancy in FET cycles [31,32]. This may be due to its immunomodulation extra-endometrial function [4] rather than the implantation process, in which the progesterone concentration in the endometrium may play a more relevant role than its serum levels [28].

The first end-point for an adequate LPS is to optimize the so-called WOI and the synchronization between embryo and endometrium. Recently, the use of the term WOI has been challenged when implantation does not occur [33]. Progesterone, with or without estrogens, can be used for LPS, as can GnRH agonists or hCG [26]. It must be noted that given its longer half-life, hCG entails a higher risk of OHSS than GnRH agonists. 

The aim of this review is to provide an overview of the uterine and extra-uterine effects of progesterone that may play a role in embryo implantation and pregnancy and to discuss the advantage of using progesterone for LPS in order to shed light on how progesterone would best be used in terms of Good Medical Practice (GMP).

Herein, we examine the evidence regarding the use of progesterone for LPS in ART, particularly in fresh ET and FET with HRT or natural cycle or natural supplemented cycle support. We will also discuss the efficacy of different routes of administration, treatment regimens, and timing to optimize decidualization of the endometrium for embryo nidation and treatment outcomes. 

## 2. History of Progesterone

George W. Corner and Allen M. Willard were the first to isolate and characterize progesterone (Pregn-4-ene-3, 20-dione) and to recognize the importance of this steroid [34,35]. However, it was at the dawn of the century that Ludwig Fraenkel who, consequent to the hypothesis of the anatomist Gustav Jacob Born (1851–1900), provided experimental proof of an endocrine function of the corpus luteum [36]. Subsequently, thanks to advances in the isolation and semiquantitative determination of the hormone (i.e., the Corner–Allen test) [37], Fraenkel purified progesterone together with his colleagues Erich Fels (1897–1981) and Heinrich Ruschig (1906–1995), along with the chemist Karl Heinrich Slotta (1895–1987). This group, known as the “Breslau group”, was dismantled because Fraenkel, Fels, and Slotta were forced to emigrate consequent to the National Socialist racial policies [36].

## 3. Biological Effects of Progesterone

Besides its paradigmatic role in reproduction, progesterone also exerts significant extra-reproductive effects via multiple non-genomic signaling pathways, including immunomodulation [4,5], inhibition of cholesterol biosynthesis [7], and neuroprotection [8,9]. Unlike synthetic progestins (19-nortestosterone) [38], natural progesterone does not exert harmful effects on atherogenesis, vasomotion [39], specific modulation of androgens, or aldosterone activities [40,41], and no teratogenic effects have been reported [42].

Progesterone can also be used to induce either amenorrhea or regular bleeding, sub-atrophy, or pre-decidual changes by modifying the dose and duration of treatment and the route of administration [43,44]. Amenorrhea can be induced by the effect of progesterone on the hypothalamus. Indeed, specific binding sites for progesterone have also been identified in the brain [45].

The latter are more numerous in the cerebral cortex, hippocampus, and hypothalamus than in other brain areas. In addition, progesterone can be synthesized and metabolized in glial cells, and some of its metabolites may react with the gamma-aminobutyric acid (GABA) receptor complex, thereby potentially eliciting hypnotic, anxiolytic or antiepileptic effects [46]. Unlike some progestins, progesterone does not affect mood [47]; rather, it may have anxiolytic [48], sedative, and hypnotic effects [9]. The hypnotic effect is not observed after vaginal administration of progesterone, irrespective of dose, while it has been reported after oral administration of relatively large doses [9,49,50]. Such clinical observations suggest differences in progesterone metabolism depending on the route of administration, and specifically on different levels in pregnanolone 5 alpha- or 5 beta-metabolites [9,50]. Therefore, the same natural steroid (progesterone) administered via different routes may differently affect the central nervous system. Thus, one route of administration may be preferred over another depending on treatment indications or expected side-effects. Many studies suggest that the effects of progesterone on the central nervous system are more likely to be related to pregnane metabolites rather than to progesterone itself. Intravenous administration of 5 beta-pregnanolone at a bolus dose ranging from 0.4 to 0.6 mg/kg induces sleep and anesthetic effects within a few seconds in men [51]. These metabolites do not act through the progesterone nuclear receptor, but through the GABA membrane receptor, and are 700–1000 times more potent than most active barbiturates in stimulating the binding of benzodiazepines or inhibiting the binding of convulsant drugs [52]. The extra-endometrial effects of progesterone were not considered relevant for reproductive function; however, the different live birth rates (LBR) observed in patients who share a similar implantation rate may be correlated to the route of administration of progesterone (vaginal or vaginal plus intramuscular progesterone administration) for LPS [31]. This observation led to the rethinking of the relevance of the extra-endometrial effects of progesterone in governing ongoing pregnancy. In fact, the discrepancies between implantation rate and LBR when progesterone is administered both vaginally and intramuscularly (IM) versus vaginal administration alone may indicate that circulating progesterone affects the immunological facilitation of trophoblast invasion and penetration into the decidualized endometrium [53].

Steroid hormones also exert genomic and non-genomic effects. While a large body of data is available regarding the genomic effects, little is known about the non-genomic effects of progesterone and its single metabolite in terms of receptor and non-genomic effects. The cellular/physiological effects of progesterone mediated by the progesterone receptor (PR) are generally not rapidly elicited because of the time required to induce the transcription of genes and translate them into protein products. In contrast, progesterone can elicit rapid, non-genomic effects in various tissues via alternative mechanisms [54]. These “non-classical” effects occur rapidly at the cell surface, thereby activating the intracellular signaling pathways by modulating putative cell surface receptors, ion channels, and cytoplasmic second messenger cascades. It is noteworthy that although these effects of progesterone are “non-genomic”, the rapid activation of cytoplasmic kinase signaling can result in both transcription-independent and transcription-dependent effects [54]. Non-genomic signaling is considered to play a major role in mediating some of the effects of progesterone, e.g., the myorelaxant effect on the myometrium [6]. The complexity by which progesterone exerts its effects on target tissues reflects the variety of mechanisms that must be modulated in the attempt to develop new therapeutic strategies [54]. For instance, some of the effects of progesterone are exerted via its metabolites (e.g., allopregnanolone or 3α,5α-tetrahydroprogesterone) [55,56,57,58]. Consequently, until the role of each metabolite in determining progesterone’s biological effect is fully characterized, it will not be easy to establish a fixed relationship between exogenous administration and the aforementioned effects. 

## 4. The Immunological Effects of Progesterone

Progesterone is used to increase the odds of embryo implantation and reduce miscarriages and the risk of premature labor [59,60]. These effects may be related to the action exerted by progesterone on the immune system in the uterus [61]. In particular, the role of progesterone as a modulator of inflammation, both systemic and intrinsic to the uterus, during human pregnancy and labor [53,59]. Progesterone signaling in the myometrium has been attributed to the suppression of myometrial contractility by hindering pro-inflammatory cytokine production. Progesterone regulates local and systemic inflammation. In addition, it appears to dampen pro-inflammatory cytokine production in peripheral blood leukocytes, which is a process that reduces T-helper subtype differentiation and proliferation. Furthermore, progesterone blocks natural killer cell degranulation and, therefore, cytolytic function. 

## 5. Metabolism

The metabolism of uterine tissue may be related to the hormonal mechanism of progesterone so as to enable decidualization in the endometrium and inhibit myometrium contraction [6,62]. The immunomodulation action of progesterone in the decidua upon trophoblast invasion has been widely studied [63,64,65]. The major metabolite in the endometrium is an unknown dihydroxy compound, while 4-Pregnen-20 alpha ol-3 is the major product of the myometrium. Six other products have been identified in the endometrium, and three have been identified in the myometrium [66]. The metabolic clearance rate (MCR^r^), plasma concentration (c), production rate (P^I^) of progesterone, and the conversion to progesterone of 20α-hydroxypregn-4-en-3-one (20α-OHP) have been determined in women during the menstrual cycle [67]. The MCR^p^ was 3110 ± 217 (SE) L/day irrespective of cycle day. The P^I^ was 0.92–2.53 mg/day on days 6–18 of the cycle and 0.78–15.6 mg/day on days 20–24. The per cent conversion of 20α-OHP to progesterone was 4.88 ± 1.10 (SE)% and the calculated transfer constant of 20α-OHP to progesterone was 0.05. The contribution of progesterone to the blood production rate of 20α-OHP on days 20–24 was between 24 and 73% [68]. The corpus luteum also secretes 5 alpha-pregnane-3,20dione (5 alpha-DHP) in an amount eightfold higher than in the follicular phase. The concentration of 5 alpha-DHP in ovarian venous plasma draining and in ovaries containing the corpus luteum was twenty-twofold higher than the concentration in plasma from the contralateral vein [69]. 

## 6. Circulating Levels of Progesterone

Circulating progesterone during the mid-secretory phase has been used as a marker of endometrial receptivity in both natural conception and ART treatment [70]. However, its predictive value continues to be matter of debate [27]. The Noyes histological criteria [18] used to establish whether the endometrium is in or out of phase are based on data about the transformative effects of progesterone in the different phases of the menstrual cycle. Therefore, there is a close connection between the effects of progesterone and endometrial adequacy required for successful embryo implantation. During the follicular phase, a high circulating progesterone level indicates premature luteinization and endometrial asynchrony [71,72,73]. This observation was confirmed in in vitro fertilization (IVF) cycles in which premature elevation of progesterone predicts a dismal prognosis [74]. Furthermore, it has been suggested that luteal progesterone values <10 ng/mL indicate an LP defect that is associated to infertility and recurrent pregnancy loss [16,75]. Fresh IVF cycles lack endogenous progesterone support in the LP, mainly because of inhibition of luteinizing hormone secretion. The administration of LPS is therefore critical for successful IVF, and progesterone is the best choice in terms of efficacy and safety [26]. 

Serum progesterone levels may also be associated with the success of FET cycles. In fact, low circulating progesterone levels resulted in lower LBRs and a higher risk of miscarriage versus higher progesterone levels [32,76,77]. It has been suggested that restoring progesterone circulating levels may be sufficient to obtain optimal LPS [78,79]. However, an excess of progesterone may be detrimental [80,81,82]. A recent meta-analysis found that circulating progesterone <10 ng/mL can be considered a threshold for a good or bad prognosis in ART [27]. Given progesterone metabolism, its levels both in the blood and in the uterus differ according to the route of administration. Progesterone serum levels after oral administration have been reported to be well below the lower limit of normal range observed in the LP [83,84] because metabolism of ingested progesterone occurs mainly in the intestine. Moreover, progesterone was found to be predominantly metabolized to 5 alpha-reduced derivatives, irrespective of the route of administration. Given the metabolic pathways elicited and the peripheral plasma levels, the vaginal route appears to be better than the oral one for hormone replacement [28]. In the extracorporeal uterine perfusion model, vaginal progesterone migrates through the uterine tissue and plateaus in the endometrium and myometrium in approximately 6 h [84]. Compared with IM administration, the vaginal route led to a higher tissue concentration of P in the endometrium but lower serum levels [28], albeit with a similar implantation rate [31]. Orally administered progesterone undergoes several metabolization steps in the gut, intestinal wall, and liver [85]. Metabolism starts in the intestine, where intestinal bacteria trigger 5 beta-reductase activity. Subsequently, the intestinal wall, which exerts 5 alpha-reductase activity, initiates conjugation of steroids with glucuronic acid. After circulation in the portal vascular systems, progesterone reaches the liver to be metabolized by liver enzymes. Liver cells in women express 5 beta-reductase and 3 alpha- and 20 alpha-hydroxylase activities, and are also able to conjugate steroids with glucuronic acid. Only a fraction of the progesterone administered eludes enzyme activity and circulates in the plasma, while most of the steroids circulate as inactive 5 beta-pregnane-3 alpha ol- 20 alpha-diol-glucuronide. High levels of circulating progesterone and pregnanolone metabolites have been described after oral administration of micronized progesterone [86]. By contrast, normal vaginal bacteria and mucosa seem devoid of 5 alpha- and 5 beta-reductases and 3 alpha- and 2 alpha-hydroxylases, and progesterone is absorbed without undergoing significant metabolic changes. Thus, oral administration of micronized progesterone increases both progesterone plasma values, as well as 5 alpha- and 5 beta-pregnanolone levels that then act directly on GABA receptors in the central nervous system. The latter process could explain the side-effects exerted on the central nervous system observed after oral administration of progesterone. By contrast, vaginal administration, which is highly effective in inducing secretory endometrium, elicits only minor changes in plasma levels of “psychotropic” metabolites [86]. Lastly, a recent systematic review and meta-analysis on patients with miscarriage symptoms was conducted to determine whether progesterone serum levels could have prognostic value for pregnancy viability [87]. The authors concluded that a single measurement of progesterone levels provides clinically useful prognostic information about pregnancy viability. More than nine out of ten patients with a progesterone level < 6.3 ng/mL are diagnosed with a non-viable pregnancy, while more than 90% of patients with a level ≥ 20–25 ng/mL (63.6–79.5 nmol/L) can have their viable pregnancy confirmed [87].

## 7. The Endometrium

Progesterone is the main actor in the endometrium saga, and estrogens are its co-stars [88]. Progesterone action on the endometrium is manifested by secretory modification [66]. The classic Noyes criteria [18], used by clinicians for the past 50 years to evaluate LP defects, is falling out of favor due to conflicting views on the timing and interpretation of endometrial biopsies [89,90]. Thanks to technological advances [91] and new data on decidual markers [21], we have improved our ability to evaluate the menstrual cycle and to identify endometrium morphological changes with greater precision than before. For instance, electron microscopy can detect pinopodes [22,92]. Moreover, we can use molecular methods to identify endometrial differentiation during the menstrual cycle [17]. All these methods concur to address the key questions regarding endometrial adequacy differentiation: is the endometrium in or out of phase, and is it adequate or not for embryo nidation? Being able to answer these questions is of paramount importance in reducing implantation failure of “good embryos”, particularly in ART programs. However, these questions remain unanswered, and we need new methods to evaluate the endometrium. 

### 7.1. Markers of Endometrial Function

Shoupe et al. [90] emphasized the need to chronologically sequence the critical changes of the menstrual cycle in a clinical perspective. They studied four menstrual cycle parameters as reference points for endometrial dating: ultrasound demonstration of ovulation, LH surge, basal body temperature shift, and the onset of menstrual flow post-biopsy. An agreement of ±2 days was found in 96%, 85%, 77%, and 65% of the samples, respectively. Using scanning electron microscopy of the uterine luminal epithelium, Martel et al. [22] and Nikas et al. [92] identified pinopodes, which are apparently involved in the mechanisms of transduction of the surface epithelium and in the exchange of fluids and low-molecular weight proteins. Progesterone stimulates the appearance of pinopodes, whereas estrogens cause their regression [22]. Pinopode development has been linked to the adhesion of blastocysts to the luminal epithelium, thereby suggesting endometrial nidation receptivity [93]. Nikas and Psychoyos [94], along with Lessey [95], identified stage-dependent changes in pinopode formation during normal and stimulated menstrual cycles. During normal menstrual cycles, the number of pinopodes peaks on days 19, 20, and 21 of a 28-day cycle [94]. In stimulated cycles using clomiphene citrate (100 mg/d) for five consecutive days, followed by hMG on days 6, 8, and 10 of the cycle and subsequent administration of hCG (5000 IU), pinopodes were observed on days 16, 17, and 18 [96]. Thus, in stimulated cycles, the timing of pinopode formation appears to occur several days earlier than occurs in spontaneous cycles, and seems to be a morphological marker of adequacy for endometrial implantation. Unfortunately, the need to use scanning electron microscopy to identify pinopodes precludes the use of this morphological biomarker in daily clinical work. The results with clomiphene citrate, and also with gonadotropins, should be tested.

The use of molecular techniques and immunohistochemistry led to the identification of other biomarkers in the human endometrium [21]. Some of them, located on the surface of the luminal epithelium, are significant factors in the embryo-endometrium apposition, adhesion, and attachment [97]. Others, located at the level of the extracellular matrix of the endometrial stroma, are significant factors during trophoblast invasion. For instance, basement membranes supporting lumen surface epithelial cells, which depend on progesterone for both formation and cyclical disintegration, constitute a barrier for embryo penetration after adhesion [21].

Adhesion molecules of the integrin category that are present during the secretory phase are biomarkers of receptive differentiation of the endometrium [95,98,99]. Another biomarker is a polypeptide growth factor known as leukemia inhibitory factor (LIF), which belongs to the family of epidermal growth factors (EGF). In the mouse endometrium, LIF is required for normal implantation. Embryos from transgenic mice without LIF expression are unable to implant, although they develop normally in vitro [100]. In women, LIF has been found in the endometrium at the theoretical time of implantation [101], with maximal expression occurring between days 19 and 25 of an ideal cycle [102]. It is conceivable that in abnormal or stimulated cycles, the expression of LIF in women may differ from natural cycles. This also applies to other markers due to advanced endometrial maturation in stimulated cycles [103]. In the adhesion phase, LIF stimulates trophoblastic differentiation (villous to invasive syncytiotrophoblasts). However, our previous attempt to use recombinant LIF (Peprotech EC Ltd., London, UK) in vivo, either by conditioning the embryo culture and/or the endometrial cavity with a constant infusion for 48 h before ET, did not significantly improve ongoing pregnancy in IVF cycles [104]. 

Other endometrial biomarkers have been analyzed in the attempt to find the key factor promoting embryo implantation, but without success [19,105]. Endometrial receptors for E2 and P4 are essential for hormonal genomic action and for the expression of some biomarkers of its receptive differentiation. Both receptors reach their maximal expression in the glandular epithelium and stroma during the late proliferative and early secretory phases [106]. After day 19, E2 and P4 receptors abruptly disappear from the glands, probably due to the effect of progesterone, while they persist in the stroma [107,108]. Other techniques based on gene expression mapping with which to recognize a receptive endometrium [107] have recently become commercially available. However, the gene expression mapping to coordinate adequate timing for embryo transfer fails to obtain the desired results [109].

### 7.2. Metabolism of Progesterone in the Human Endometrium

Metabolism of progesterone in the human endometrium has been described in both whole tissue and cellular fractions of a normal endometrium [110]. Using thin-layer chromatography, Casey et al. [88] studied progesterone metabolism in stromal and gland cells in culture. The most abundant metabolite in both cell components was 3 beta-hydroxy-5 alpha-pregnan-20-one (70%), followed by 5 alpha-pregnane-3,20-dione (15%), and 3 alpha-hydroxy-5 alpha-pregnan-20-one (10%). It was established that a small amount of progesterone is metabolized to 5 alpha-pregnane-3 alpha/3 beta,20 alpha-diols and to 3 beta,5 alpha-dihydroxy-5 alpha-pregnan-20-one. The metabolism of progesterone in cultured endometrial cells occurs rapidly; 70% of progesterone is metabolized in 8 h, and 90% in 24 h. The speed and extent of the metabolism of natural progesterone should be taken into account when it is used exogenously [110]. In 1970, Sweat and colleagues reported that 4-pregnen-20 alpha-ol-3-one and allopregnanedione are the predominant products of the endometrium [66].

To investigate the endometrial and myometrial metabolism of progesterone after vaginal application, Bulletti et al. [111] used the following metabolites of progesterone: 20α-hydroxy-4-pregnen-3-one (20-DHP), 5 alpha-pregnane-3,20 dione, and 5 beta-pregnan-3,20 dione, 5 alpha-Androstane-3 alpha-allopregnanolone. Using the extracorporeal uterine perfusion system [111], two sets of experiments were conducted: cold and radioactive progesterone. Progesterone was applied on the rim of the remaining vaginal tissue attached to the uterus. Samples of endometrial and myometrial tissue and venous outflow were obtained after 3, 6, 9, 12, 24, and 48 h, using at least three uteri per time interval. Progesterone and P4 metabolites present in the venous outflow and tissue samples were taken as indicators of regional and whole organ metabolism of progesterone (Table 1). Progesterone is metabolized by the human uterus to three primary metabolites, a process that may cause all or part of the effects exerted by progesterone on the myometrium via the non-genomic properties of these metabolites [112] during the short time between stimuli and contractions. However, this issue remains controversial [113]. 

**Figure 1:**
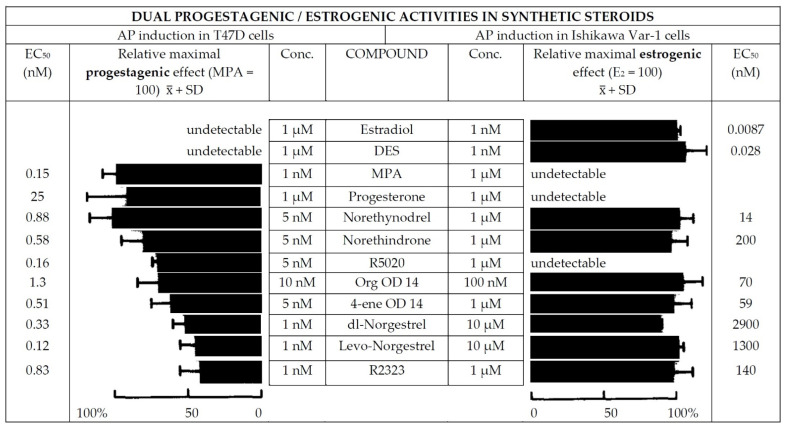
Progestagenic and estrogenic activity of various progestins. Reprinted from Ref. [114]. Copyright 1994, with permission from Elsevier.

Progesterone and progestins exert both progestagenic and estrogenic effects on endometrial cell lines. The choice between natural and synthetic progesterone depends on the therapeutic goal, namely (i) adequate endometrial differentiation obtained with HRT for FET or (ii) antiproliferative activity on the endometrial tissue during HRT in post-menopause. In an elegant in vitro study of endometrial cells, Markiewicz and Gurpide [114] demonstrated that C19 derivatives exert both estrogenic and progestagenic effects, whereas C21 derivatives exert mainly progestagenic effects (Figure 1) [114]. 

## 8. The Myometrium

Progesterone affects the myometrium by inhibiting muscle contraction [66]. In the non-pregnant uterus, estrogens are myo-contractant and progesterone is myo-relaxant [6]. Uterine contractility has a specific pattern during the menstrual cycle that may be related to gamete and embryo transportation through the genital tract before conception and implantation [62]. 

**Figure 2:**
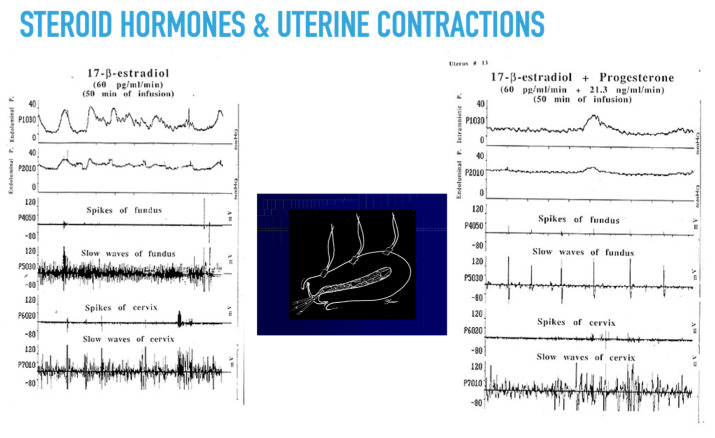
Electromechanical activities of human uteri during the extracorporeal perfusion of human uteri. Silver-silver electrodes recorded the electrical signals, and intraluminal pressure probes detected the pressure variations [6].

Dysfunction of uterine activity can contribute to subfertility. In ART cycles, implantation is inversely related to the number of uterine contractions at the time of ET [115,116]. Women with endometriosis also have abnormal uterine contractility [117], and vaginal parturition reduces the risk of recurrences of endometriosis [118]. The time lapse between steroid stimulation and myometrial response suggests that the myorelaxant effect is caused by non-genomic effects (Figure 2) [6], although this is a much-debated issue [113]. 

## 9. Progesterone Supplementation: Who, When, and What?

### 9.1. The Rationale behind LPS in Fresh ART Cycles

**Figure 3:**
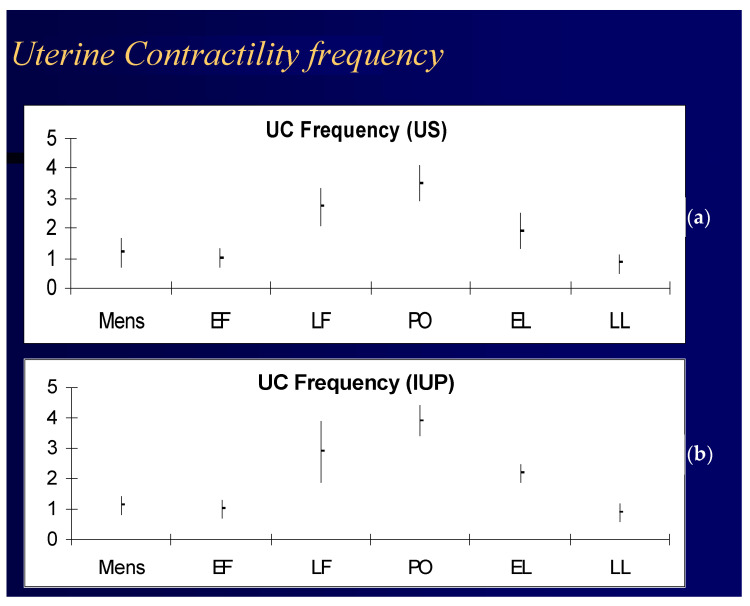
(**a**) Frequency of uterine contractions detected in vivo in a cohort of menstruating women during the menstrual cycle detected with ultrasound (US) and Intrauterine Pressure detection (IUP); (**b**) Timings detected were menstrual cycle (mens), early follicular (EF), late follicular (LF), peri-ovulatory (PO), early luteal (EL), and late luteal (LL) phase of normal menstrual cycles. Adapted with permission from Ref. [62]. Copyright 2000, European Society of Human Reproduction and Embryology.

Adequate LPS is essential during IVF and ICSI in order to improve implantation and pregnancy rates. This can be achieved by substituting deficient LH with GnRH agonists or hCG or by directly using progesterone with or without estrogens [119]. Notably, hCG increases the risk of hyperstimulation and multiple pregnancy due to its longer half-life. After ovulation, the LP of the menstrual cycle continues until the next menstruation. Remnants of the ovulated egg in the ovary form the *corpus luteum* (or “yellow body”) that produces estrogens and progesterone. The endometrial preparation for embryo nidation starts in the proliferative phase and extends throughout the LP. Estrogens induce PR synthesis. Progesterone stimulates differentiation of the endometrium of the uterus to prepare for implantation [120]. The LP begins on the day of the LH surge and ends approximately 14 days later. In natural cycles, secretory transformation of the endometrium (the so-called pre-decidualization phase) enables embryo implantation [121], which occurs six days after fertilization. Around three to five days pre-implantation, the embryo migrates through the tubal-lumen cavity of the uterus thanks to the myometrial activity that is sustained by estrogens and progesterone (Figure 3) [62]. After implantation, the trophoblastic tissue of the placenta secretes hCG, thereby maintaining and stimulating the corpus luteum to produce estradiol and progesterone [122]. Progesterone production by the corpus luteum is required to maintain pregnancy until the placenta takes over the production of steroid hormones at approximately seven weeks. 

In fresh ET ART cycles, the woman’s pituitary gland is desensitized with GnRH analogs so that the ovaries can be stimulated in a controlled manner in order to obtain the highest number of mature eggs possible; this in turn induces an LP defect because the corpus luteum is unable to produce sufficient progesterone. Luteolysis is caused by a high concentration of steroids secreted by a large number of corpora lutea during the early LP, which in turn inhibit LH release via negative feedback to the hypothalamus [123].

The LP must be supported in fresh ART cycles because low progesterone concentrations may reduce the chances of implantation. Luteal phase support is achieved by administering either progesterone, hCG (to stimulate progesterone production), or GnRH agonists (to stimulate GnRH production by the hypothalamus, which in turn stimulates the release of LH). GnRH agonists are thought to restore LH levels, thereby supporting the LP in a physiological manner [124]. Progesterone for LPS counteracts the endometrial proliferation of estrogens and provides for pre-decidualization of this tissue, which is a prerequisite for receptivity of embryo nidation and for the establishment and maintenance of pregnancy [1,2,125]. Progesterone supplementation has proved to be effective for LPS [31,126,127,128,129,130,131,132].

### 9.2. The Rationale for LPS in ART Cycles with Frozen Embryo Transfer

Although Wong et al. [133] reported that a freeze-all strategy does not improve cumulative ongoing pregnancy rates, FET is increasingly being used to treat infertility. According to the International Committee for Monitoring ART, of the 1,955,908 ART cycles performed in 2017, nearly 600,000 were classified “FET” with a delivery rate of 26.1% [134,135]. The increased use of FET [136,137] stems from the extensive use of cryopreservation for pre-implantation genetic testing [138,139,140], single ET, and freeze-all policies [141] to avoid severe OHSS [142] and maternal and fetal risks associated with multiple pregnancies [138]. 

In the FET HRT cycles using estradiol and progesterone substitution cycles, there is no corpus luteum, and therefore estradiol is not produced endogenously. In HRT cycles, identification of the endometrial preparation before ET is crucial to optimize the chances of live birth [143]. Exogenous progesterone replacement is required to program endometrial preparation [134,144,145], and treatment should be continued until the switch to trophoblast production. The implantation rate of FET with natural cycles is significantly higher than for FET with HRT [146], thereby suggesting that progesterone serum levels may be representative of good endometrial preparation [147].

The vaginal and intramuscular routes are the ones most widely used for progesterone administration. The oral route is generally not used for LPS because of poor bioavailability and poor ART outcomes [148,149,150]. Daily injections of intramuscular progesterone are painful and carry the risk of abscessual complications; consequently, the vaginal route is generally preferred for ART patients [151,152,153]. However, the gold standard of progesterone replacement for FET has yet to be established. In 2010, a Cochrane review of four randomized controlled trials (RCT) comparing the vaginal route with intramuscular routes found no statistically significant differences in live birth, clinical pregnancy, or pregnancy loss rates [145]. Conflicting results have been reported in retrospective studies comparing vaginal and intramuscular progesterone for FET [154,155,156,157]. Notably, one retrospective study reported a significantly higher LBR using IM progesterone once every third day, in addition to daily vaginal progesterone versus daily vaginal progesterone alone [27,158]. 

Given the increasing use of preimplantation diagnosis and the therapeutical tools now available, the implantation of “good” embryos in the prepared endometrium remains the major limiting factor for successful ART programs. In fact, the overall implantation rate varies from 51.1% in women <35 years of age to 8.6% in those over the age of 42 [86,159]. Notably, the embryo’s aneuploidies are the most limiting factor in the implantation process and possibly also in an ongoing pregnancy [160,161], with a potential improvement of only 5% to 15% over other interventions. Progesterone affects the endometrium by reducing endometrial proliferation, by developing more complex uterine glands, by collecting energy in the form of glycogen, and by providing greater uterine blood vessel surface area that is suitable for supporting a growing embryo. However, researchers have not yet been able to identity a key control phenomenon within this sequence [162]. Although the classical mechanism by which progesterone elicits its actions is the regulation of gene expression, progesterone also elicits its effects via non-genomic mechanisms [54]. Nevertheless, the exact role of non-genomic mechanisms remains unknown. However, it is well established that the metabolic pathway of progesterone varies depending on the route of its administration [86] and that each metabolite exerts genomic and non-genomic effects. Consequently, the different administration routes of progesterone provide adequate LPS, albeit via different mechanisms in the endometrium. In fact, exogenous IM and vaginal progesterone are remarkably effective in triggering the full array of endometrial changes that normally occur in the LP of the menstrual cycle [28,163,164]. This finding differs markedly from those obtained with oral progesterone, that were, nevertheless, incomplete [150]. Various studies on the progesterone requirement for endometrial receptivity and embryo implantation found that vaginal (but not oral) progesterone consistently induces an “in phase” late luteal endometrium [143,165,166,167]. These findings explain why the vaginal route of administration became the routine form of luteal support. When vaginally administered, progesterone may affect uterine function through both its specific pharmacokinetic transit time (first uterine pass) [84] and its specific local metabolism [110]. 

## 10. Variables to Be Considered in the Relationship between Progesterone Production/Administration by Different Routes and Their Biological Effects

When attempting to establish a relationship between progesterone production and its efficacy, whether endogenously or exogenously administered by different routes, some important (albeit often neglected) variables must be considered, namely:

- The production rate, which also depends on the MCR, defined as the volume of blood irreversibly cleared of a substance over time, which in turn supports the serum concentration [68]. Notably, the MCR of progesterone may change depending on exercise, body temperature, and other factors;
- The transport of circulating progesterone. Only free genomically bio-active steroid hormones (corresponding to about 2%) reach the endometrial cell compartment, while the remaining 98% are protein-bound and ineffective [28,111,168,169,170,171,172,173,174]. Steroid hormone transportation from blood to the tissue compartment has been considered a permanent parameter. However, the ultrafilters of basement membranes (BM) located around microvessels and glands (with micropores the size of 75,000 Amstrong) in the endometrium are not an on/off switch; rather, they progressively develop during cycles. Therefore, their function as an ultrafilter may also be progressive [169], and cyclical BM synthesis and degradation also depend on progesterone. In other words, BMs serve to separate tissue compartments in which steroids freely circulate between compartments, while circulating protein-bound steroids are blocked by BM pores [21];
- Metabolites in blood and tissue produced by progesterone vary depending on the route of administration, thereby inducing different effects on target tissues. For instance, the metabolism of oral progesterone differs from non-oral administration because of the first liver pass effect [68,174,175,176] and the first uterine pass effect [112]. As a consequence, different routes of administration have different effects on endometrial pre-decidualization [112];
- The genomic and non-genomic effects of progesterone that differ in terms of duration of the signal-response [54] influence ongoing pregnancy;
- Uterine contractility, with estrogens as myocontractant and progesterone as myorelaxant [6,62], has been widely studied without, however, reaching any conclusive results. The myorelaxant effect exerted on uterine musculature is associated to the myocontractant effects of estrogens, thereby participating in the transport of both the gamete and embryos within the genital tract during the ovarian cycle and embryo nidation [6,62].

## 11. Routes of Administration

Progesterone, including micronized progesterone and synthetic progestogens such as dydrogesterone [112], can be administered by different routes. In the pioneer substitution cycles designed for donor egg ART, progesterone was administered by IM injections of oil-based solutions [143], the only route possible given the lipophilic characteristics of progesterone. Oral progesterone was not a viable option because, while nearly 100% is absorbed in a micronized form, it is almost entirely metabolized during the first liver pass. 

**Figure 4:**
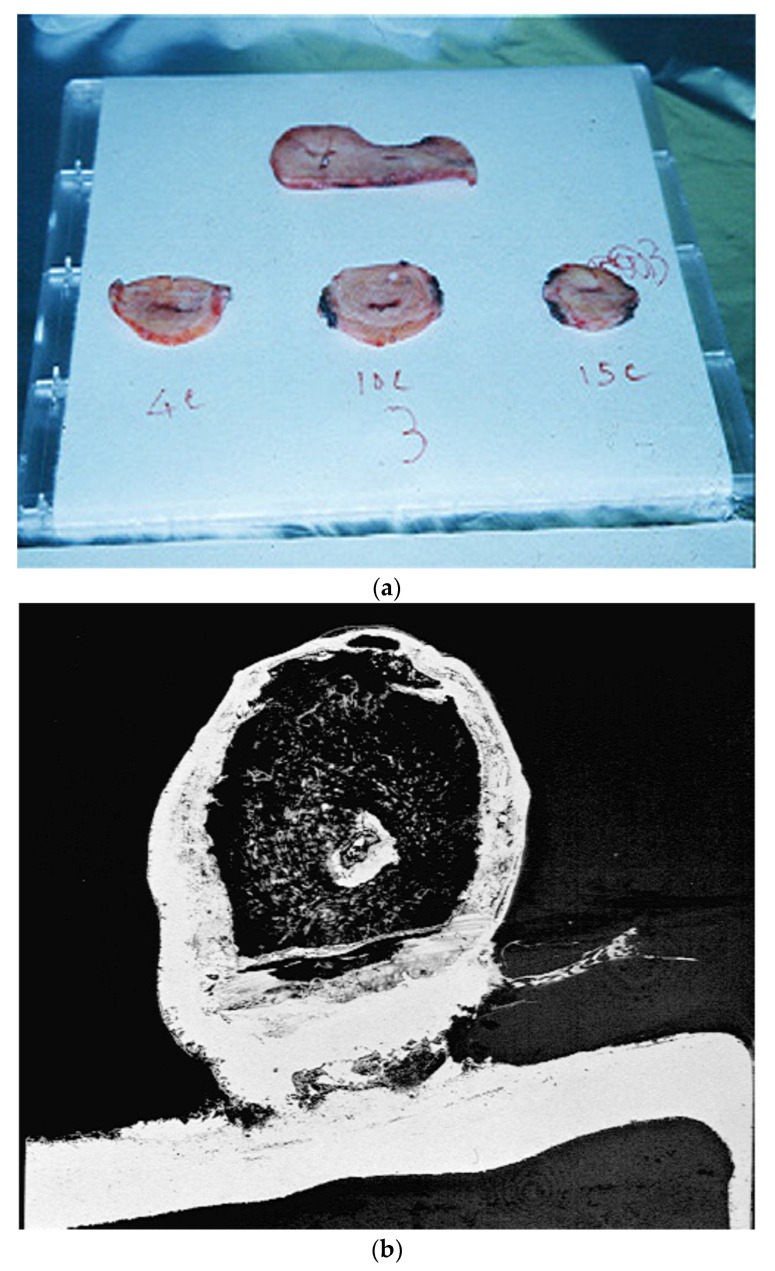
(**a**) The uterine slices after application into the vaginal collar of an extracorporeally perfused human uterus of radiolabeled progesterone (3H-P), used as a test substance, and 14C butanol, used as free diffusible reference substance, to calculate the P uterine pharmacokinetics and metabolism of the uterus; (**b**) The uptake of progesterone into the myometrium and endometrium after radiolabeled vaginal P application by autoradiography.

Unlike oral progesterone, vaginal progesterone is well absorbed and conveyed to the endometrium. In fact, radiolabeled progesterone administered in an extracorporeal perfusion of the human uterus ex vivo effectively traveled from the vaginal cuff to the uterine corpus [84]. Figure 4 shows the radioactive accumulation at a steady state of 3H-P extracted from uterine tissue [84] during the extracorporeal perfusion of human uteri [111]. These data have been confirmed in vivo [177]. Hence, vaginal administration appears to be the only alternative to painful IM injections. However, in a recent study, all routes of progesterone administration were equally effective in achieving satisfactory mean serum progesterone levels at day five (10 ng/mL) [27,31,32]. On day five, the mean ± standard deviation of serum progesterone levels after vaginal, subcutaneous, and intramuscular administration were 14.6 ± 5.5, 47.9 ± 22.3, and 60.3 ± 65.5 ng/mL, respectively (*p* = 0.032 across routes) [178].

### 11.1. Progesterone Shots or Injections (Progesterone in Oil) versus Vaginal Progesterone 

Progesterone in an oil-based solution can be injected directly into a muscle (usually the buttocks) once a day and results in high levels of circulating progesterone in the bloodstream [28]. This was the first method used and is well-recognized to be beneficial for IVF with fresh ET and HRT for FET. Side-effects may include allergic reactions, inflammation and pain at the injection site, and difficulty in walking or sitting. Progesterone may also be administered by subcutaneous injection [179].

Unlike standard IM regimens, vaginal micronized progesterone enhances progesterone delivery to the uterus and results in a synchronous secretory endometrium, as histologically demonstrated in agonadal women preparing for embryo donation [28]. However, a recent RCT found a significantly higher rate of miscarriages after vaginal-only progesterone replacement versus IM progesterone in HRT for FET [31], which suggests that above a certain threshold, serum progesterone levels improve the occurrence and maintenance of pregnancy in FET cycles. Indeed, the same study found that vaginal progesterone supplemented with IM progesterone every third day resulted in a significant increase in LBR in IVF cycles versus vaginal progesterone alone, despite no difference in implantation rate [31]. Furthermore, progesterone IM administration every three days in addition to vaginal administration was equally effective, as was daily IM administration, thereby suggesting an alternative regimen with fewer injections. Based on these findings, one may hypothesize that the extra-uterine effects of IM progesterone or its metabolites are crucial in maintaining pregnancy. However, this issue remains controversial. In intrauterine insemination or sexual intercourse conception, it is uncertain whether LPS with vaginal or IM progesterone increases pregnancy rates and decreases miscarriages, although there is evidence that progesterone may increase clinical pregnancy compared to placebo in all types of ovarian stimulation [180]. 

Vaginal administration of progesterone (Table 2), which is highly effective in inducing a secretory endometrium, elicits only minor changes in the plasma levels of “psychotropic” metabolites [49]. Given the metabolic pathways and the peripheral plasma levels obtained, the vaginal route appears to be more effective than the oral route for hormone replacement [181]. Progesterone can be administered vaginally by a gel or cream, which results in high endometrial concentrations by bypassing the first-pass effect through the liver [182], and it may also reach higher endometrial concentrations consequent to the first uterine pass effect [28,84]. 

### 11.2. Progesterone Capsules (Used Orally)

Oral progesterone supplementation is considered less effective than other administration routes in women undergoing ART. Oral progesterone can result in side-effects such as nausea, bloating, drowsiness, and irritability. Progesterone levels attained after oral administration have been found to be well below the lower limit of the normal range observed in the LP [83]. Metabolism of ingested P occurs mainly in the intestine and liver. The absorption, metabolism, and clearance of progesterone from the peripheral circulation have been investigated after oral administration of 100 mg daily for five consecutive days [183]. Plasma concentrations of progesterone peaked within four hours of ingestion of the last dose, with a range of 22.11–34.18 nmol/L; 696–1077 ng/100 mL. This range is comparable to that observed during the mid-luteal phase of the ovarian cycle. The surge in values lasted six hours, and progesterone concentrations remained high for at least 96 h. Of the three metabolites studied, the plasma concentrations of pregnanediol-3 alpha-glucuronide were higher post-treatment than for the other metabolites. In fact, the peak values ranged from 1097 nmol/L (54.9 microgram/100 mL) to over 2000 nmol/L (100 microgram/100 mL), which was the upper limit of the assay used. Concentrations of 17-hydroxyprogesterone were the ones that least raised the peak values, ranging from 4.32 to 9.68 nmol/L (143–319 ng/100 mL). The plasma profile of 20 alpha-dihydroprogesterone most closely approximated that of progesterone, although the range of maximal values was lower (7.11–16.06 nmol/L; 228–514 ng/100 mL). Plasma concentrations of estradiol were unchanged by progesterone. Therefore, oral progesterone may play a therapeutic role [183], albeit considering the aforementioned limitations [83].

For how long the LP should be supported remains an open issue. A meta-analysis found no differences between short (14 days) versus long (up to 12 weeks) LPS (in terms of clinical pregnancy) [26]. No evidence supports the use of progesterone for LPS after the eighth week of pregnancy, namely when the of corpus luteum/placenta progesterone production switch occurs [26]. However, as discussed above, progesterone may play a role in preserving ongoing pregnancy in non-genetic threatened abortion [87,184], possibly due to both extra-endometrial immunological protective effects and its own myorelaxant action.

## 12. Discussion

With its wide range of biological functions, progesterone is the star of human reproduction. By supporting the secretive phase of the menstrual cycle, progesterone optimizes endometrial adequacy for embryo nidation in vivo and in vitro. Insufficient progesterone production leads to luteolysis and LP defects that reduce the chances of pregnancy by causing recurrent implantation failure (RIF), which may occur at earlier stages of implantation or later when pregnancy has been established [1,2,26,145]. In ART, the use of GnRH protocols that block the pituitary gland may induce premature luteolysis, which results in significantly decreased pregnancy rates [185]. In this context, LPS with progesterone has become standard practice in fresh ET cycles. Vaginal or intramuscular progesterone, alone or in combination, are the most effective routes of administration and are equally effective [26]. Recent studies suggested that the value of mid-secretive phase circulating levels of progesterone for successful implantation and live birth after FET should be reconsidered [31,61]. Indeed, progesterone serum levels >10 ng/mL during INF programs resulted in a better prognosis. Recent systematic reviews have established that while intratissular progesterone concentration is relevant to improve implantation rates [28,84], the circulating levels of progesterone at mid-secretory phase affect LBR, probably due to the extra-endometrial (probably immunomodulation) role played by progesterone. Such findings emphasize a still-unclarified extra-endometrial function of progesterone. Studies of FET HRT reported late pregnancy complications in women treated with progesterone for LPS [186]. The latter finding suggests that the corpus luteum may exert a protective effect on pregnancy, possibly through the immunological modulatory effects (e.g., progesterone-induced blocking factor) that have yet to be clarified [164]. Embryo attachment to the endometrial epithelium promotes the formation of multinuclear syncytiotrophoblasts from the trophectoderm, which then breach the epithelial layer [187]. The following questions remain unanswered: is there an endometrial-promoting factor, and if so, is it related to progesterone priming? We still do not completely understand the non-genomic effects of progesterone that may explain, in part, these extra-endometrial protective effects.

Vaginal, IM, and subcutaneous routes are the ones most often used for progesterone supplementation, with vaginal as a standard route. This review confirms the beneficial role of progesterone in LPS of IVF in fresh ET cycles, with no differences in terms of IR between vaginal and IM or subcutaneous administration. Furthermore, in FET HRT, vaginal progesterone tablets alone versus vaginal plus IM have been reported in an RCT [31] to result in a similar IR but a lower LBR. More studies are needed to confirm this result, as this study has been criticized with respect to differential timing of the onset of progesterone administration in its 3 different arms. Oral progesterone derivative LPS is not considered a first-line option because of the great variability in endometrial and clinical effects [188]. In GMP, the role of the corpus luteum in FET cycles must be considered because of the lack of pregnancy complications in the third trimester [31,189].

In LPS for natural and modified (hCG-triggered) natural FET, the optimal timing to mimic the corpus luteum function is to start progesterone 36 h after the onset of the LH surge or 36 h after hCG administration, and to program blastocyst transfer after five full days of progesterone supplementation [189].

Progesterone is also involved in the protection of ongoing pregnancy. In fact, in the management of threatened miscarriage, NICE now recommends offering “vaginal micronized progesterone 400 mg twice daily to women with an intrauterine pregnancy confirmed by a scan, if they have vaginal bleeding and have previously had a miscarriage”, and “if a fetal heartbeat is confirmed continue progesterone until 16 completed weeks of pregnancy” [190]. NICE recognized that this was an off-label use of vaginal micronized progesterone [190].

## 13. Future Directions

The effects of progesterone on systemic and uterine cyclical transformation that lead to embryo implantation, ongoing pregnancy, and live birth depend on the following: its production, metabolism, the effect of a single metabolite, genomic and non-genomic tissue responses, and lastly, on the route of administration when exogenously administered. Embryo implantation and ongoing pregnancy are largely determined by the genetic viability of the embryo [191]. In addition, an adequate endometrial preparation by endogenous progesterone produced by the corpus luteum and/or exogenous progesterone also determines successful implantation in the measure of 5–15% in each cycle [160,161]. The role of progesterone becomes significant for couples undergoing three to four aspirations of ovarian follicles in their IVF attempts. 

Future studies should aim to:

- Establish both morphological and biochemical endometrial adequacy for embryo nidation using known validated parameters and the correlation with euploid embryo implantation;
- Establish the validated endometrial features required for embryo nidation by using different routes of administration, including associations of routes and related implantation rates as well as live birth rates after euploid ET;
- Compare different types and doses of vaginal progesterone administration with respect to efficiency, safety, and patient reported outcomes in the context of LPS after ovarian stimulation with or without IUI, and in the context of ART treatments, prevention of miscarriage and prevention of preterm birth;
- Evaluate new routes or associations of routes of progesterone administration, including intrauterine progesterone administration;
- Establish the role of circulating progesterone in implantation, progression of trophoblast invasion, and LBR after euploid ET;
- Reinforce the evidence that the corpus luteum plays a protective role in late-pregnancy complications in FET by using HRT.

While scientists endeavor to address these future challenges, as clinicians we should commit to:

- Promote and support studies to explore novel issues regarding the role of progesterone in achieving successful IVF programs [192,193]. Indeed, because of the cost and time required to conduct each type of study, the literature is overloaded with systematic reviews and meta-analyses, whereas there is almost a complete lack of new ideas and approaches. We need to pursue new ideas rather than depend on the ideas of others;
- We must reverse the mental order of the approach to this research. Given that physiology never provides evidence of a 1:1 ratio between the transferred embryo and the healthy baby that is born, we should not consider physiology as the gold-standard objective of our scientific commitment. The goal of research aimed at live birth must be more ambitious and supraphysiological. As Heraclitus reminds us, “Whoever does not try the impossible never reaches it”. Moreover, if you see only the stars above you, you will never be a good astronomer.

## 14. Key Messages

- Progesterone is an ovarian steroid produced by the granulosa cells of follicles after the LH peak at mid-cycle.
- Its main biological role is to prepare the endometrium for embryo nidation and support an ongoing pregnancy.
- Progesterone exerts extra-endometrial biological functions on the central nervous system and on uterine contractility of both pregnant and non-pregnant uteruses.
- The functions of progesterone depend on genomic and non-genomic actions: the former are well known, whereas the latter actions have yet to be fully clarified.
- Exogenous progesterone is administered via oral, vaginal, intramuscular, and subcutaneous routes. The lipophilic nature of progesterone precludes a transdermal formulation.
- The different routes of administration elicit different patterns of metabolites that exert different biological effects, thereby exerting different effects on the endometrium.
- Progesterone is responsible for a large number of morphological and biochemical changes of the endometrium, which optimize the endometrial differentiation in close synchronization with embryo differentiation.
- In IVF programs, progesterone serum concentration > 1.8 ng/mL at the time of trigger is an indication of delayed ET because of dissociation in the endometrial-embryonic synchronization.
- Progesterone is mainly used to prepare the endometrium for fresh ET cycles after IVF and in FET as HRT or natural supplemented cycles.
- Considering that the implantation of a good embryo is the gold standard in ART, the contribution of an adequate progesterone supplementation in endometrial preparation is estimated to be around 15%.
- The vaginal route of progesterone supplementation is considered the standard treatment because it is less painful and has higher compliance than the IM route, and it is equally efficient.
- Progesterone concentration in the endometrial tissue is higher after vaginal administration than after IM or subcutaneous administration, despite lower serum levels.
- The use of progesterone is recommended in the treatment of patients with threatened abortion.
- There is no convincing evidence to support the use of vaginal progesterone in order to prevent recurrent preterm births or improve perinatal outcomes in singleton gestations in women with a history of spontaneous preterm births [194].

## Figures and Tables

**Table 1:** Progesterone is metabolized in the human uterus into three primary metabolites, which may carry out all or part of progesterone effects on the myometrium. Adapted with permission from Ref. [112]. Copyright © 2001 American Society for Reproductive Medicine. Published by Elsevier Inc. All rights reserved.

	**% Endometrial Steroids during Perfusion at Time Intervals after P4 Administration**					
**Steroids**	**3 h**	**6 h**	**9 h**	**12 h**	**24 h**	
% P	485.21	83.36	84.82	91.56	93.36	
% 20 alpha	1.53	2.18	2.05	1.90	2.19	
%	−14.79	−16.64	−15.18	−8.44	−6.64	
% 5 alpha	4.45	5.13	5.73	3.34	1.73	
% allo	8.81	9.33	7.40	3.20	2.72	
	**% Myometrial steroids during perfusion at time intervals from P administration**					
**Steroids**	**3 h**	**6 h**	**9 h**	**12 h**	**24 h**	**48 h**
% P	89.0	86.6	79.9	81.9	83.3	84.1
% 20 alpha	3.4	3.5	3.1	3.5	3.0	3.2
%	−11.0	−13.4	−20.1	−18.1	−16.7	−15.9
% 5 alpha	2.7	3.7	7.1	6.6	5.6	6.1
% allo	4.9	6.2	9.9	8.0	8.1	6.6

**Table 2:** Vaginal progesterone preparations available for ART.

**Vaginal gel**	Available in prefilled applicators, the vaginal gel coats the walls of the vagina, thereby resulting in a controlled, steady release of progesterone. In IVF, it is used once/twice daily. Vaginal progesterone gel is approved by the authority control agencies for ART in fresh ET and for up to 12 weeks of pregnancy. Progesterone gel twice daily is also indicated for progesterone replacement in donor egg recipients and FET.
**Vaginal suppositories**	Produced by specialized fertility pharmacies, usually using cocoa-butter. The suppositories are placed into the vagina two or three times a day and they dissolve over time. Vaginal suppositories are safe and effective, but are not approved for fertility treatments by the FDA.
**Vaginal tablets or inserts**	The tablets are inserted into the vagina two or three times daily using a disposable applicator. Vaginal tablets are FDA-approved for women who need progesterone supplementation.
**Progesterone capsules (used vaginally)**	Progesterone capsules used vaginally, instead of orally, prevent side-effects. They also help to boost progesterone absorption. Oral progesterone capsules for vaginal use are not approved by regulatory agencies.

## Data Availability

Not applicable.

## Abbreviations

ART	Assisted Reproductive Technology
BM	Basement membranes
EF	Early follicular
EGF	Epidermal growth factors
EIM	European IVF-monitoring
EL	Early luteal
ER	Endometrial Receptivity
ESHRE	European Society of Human Reproduction and Embryology
FET	Frozen Embryo Transfer
GABA	Gamma-aminobutyric acid
GMP	Good Medical Practice
HRT	Hormone replacement therapy
LBR	Live birth rates
LF	Late follicular
LIF	Leukemia inhibitory factor
LL	Late luteal
LP	Luteal phase
LPS	Luteal phase support
OHSS	Ovarian hyperstimulation syndrome
PO	Peri-ovulatory
PR	Progesterone receptor
RIF	Recurrent implantation failure
US	Ultrasound
WOI	Window of Implantation
